# Sphenopalatine ganglion stimulation for cluster headache, results from a large, open-label European registry

**DOI:** 10.1186/s10194-017-0828-9

**Published:** 2018-01-18

**Authors:** Mads Barloese, Anja Petersen, Philipp Stude, Tim Jürgens, Rigmor Højland Jensen, Arne May

**Affiliations:** 1 Department of Clinical Physiology, Nuclear Medicine and PET, Rigshospitalet-Glostrup, Nordre Ringvej 57, 2600 Glostrup, Denmark; 2 Danish Headache Centre, Department of Neurology, Rigshospitalet-Glostrup, Nordre Ringvej 57, 2600 Glostrup, Denmark; 3 Schmerztherapie, Geriatrie und Palliativmedizin, Bochum, Kurt-Schumacher-Platz, 11-12 44787 Bochum, Germany; 4 Department of Neurology, University Medical Center Rostock, Gehlsherimer Str. 20, 18147 Rostock, Germany; 5 Department of Systems Neuroscience, Universitäts-Klinikum Hamburg-Eppendorf, Martinistraße 52, 20246 Hamburg, Germany

**Keywords:** Cluster headache, Sphenopalatine ganglion, Neurostimulation, Neuromodulation, Long term effectiveness

## Abstract

**Background:**

Cluster headache (CH) is a disabling primary headache disorder characterized by severe periorbital pain. A subset of patients does not respond to established pharmacological therapy. This study examines outcomes of a cohort of mainly chronic CH patients treated with sphenopalatine ganglion (SPG) stimulation.

**Methods:**

Patients were followed in an open-label prospective study for 12 months. Ninety-seven CH patients (88 chronic, 9 episodic) underwent trans-oral insertion of a microstimulator targeting the SPG. Patients recorded stimulation effect prospectively for individual attacks. Frequency, use of preventive and acute medications, headache impact (HIT-6) and quality of life measures (SF-36v2) were monitored at clinic visits. Per protocol, frequency responders experienced ≥ 50% reduction in attack frequency and acute responders treated ≥ 50% of attacks. HIT-6 responders experienced an improvement ≥ 2.3 units and SF-36 responders ≥ 4 units vs. baseline.

**Results:**

Eighty-five patients (78 chronic, 7 episodic) remained implanted and were evaluated for effectiveness at 12 months. In total, 68% of all patients were responders, 55% of chronic patients were frequency responders and 32% of all patients were acute responders. 67% of patients using acute treatments were able to reduce the use of these by 52% and 74% of chronic patients were able to stop, reduce or remain off all preventive medications. 59% of all patients were HIT-6 responders, 67% were SF-36 responders.

**Conclusions:**

This open-label registry corroborates that SPG stimulation is an effective therapy for CH patients providing therapeutic benefits and improvements in use of medication as well as headache impact and quality of life.

**Electronic supplementary material:**

The online version of this article (10.1186/s10194-017-0828-9) contains supplementary material, which is available to authorized users.

## Background

Cluster headache (CH) is a primary headache manifesting as unilateral attacks of severe pain with increased cranial parasympathetic outflow lasting 15–180 min [1]. Attacks may occur up to eight times per day and it has been described as one of the most painful sensations humans can experience. CH exists as episodic CH (eCH), where attacks occur in clusters lasting weeks-months separated by attack-free intervals > 1 month, and chronic CH (cCH) with no periods of remission lasting > 1 month for > 1 year. Around 85% of patients suffer from the episodic form but CH is by no means a static condition and patients may transition between the two forms. There are no known differences in pathophysiology and in both eCH and cCH impairment is high with reduced quality of life [2] and high direct and indirect costs [3].

Traditional treatment can be divided into acute and preventive strategies. Acute therapies consist of oxygen and injectable or nasal triptans taken as early as possible during the individual attacks. Preventive therapies are taken daily during the cluster period, or in cCH continuously, and consist of verapamil or lithium [4]. With up to eight attacks/day use of acute medications may exceed recommendations, and often doses of preventive medications higher than recommended are necessary, increasing the risk of side-effects [5]. To complicate management further, around 1% of patients are medically refractory [6]. Invasive neurostimulation, including deep brain and occipital nerve stimulation, is usually reserved for these patients. Consequently, the literature consists of reports of relatively small populations [7].

It is desirable to develop targeted treatments with no systemic side effects and no safety issues with repeated use. Sphenopalatine ganglion (SPG) stimulation using an implantable microstimulater (Pulsante™) has been evaluated in a randomized, controlled trial where 68% were therapeutic responders [8]. These results were subsequently confirmed in a 24-month follow-up study [9, 10].

The aim of this study is to evaluate the effectiveness of SPG stimulation through 12 months in an open label setting, in a large population including cCH and a limited number of eCH patients. As opposed to the previously mentioned forms of neurostimulation, ONS and DBS, SPG stimulation elicits both an acute and preventive effect which this study was specifically designed to capture.

## Methods

The Pathway R-1 registry study is a post-market registry of the Pulsante™ SPG Microstimulator System (Autonomic Technologies, Inc., Redwood City, California, USA) to confirm safety and long-term effectiveness of SPG stimulation in a large population, where patients underwent trans-oral insertion of this SPG microstimulator [11, 12].

### Patient selection

Selection criteria for this registry were patients who met CH criteria per CE -labeling for the Pulsante Microstimulation System. Patients should have cluster headaches for a minimum of 16 weeks (investigator opinion) and the ability to provide written informed consent. Exclusion criteria included changes in preventive medication in the month prior to enrollment and patients who have bony facial deformities, inappropriate surgical anatomy or have had facial surgery that would prevent the proper placement of the Pulsante Microstimulator. Given that previous studies established a good safety profile it was not a requirement that patients were medically refractory per established criteria [6], however, most had tried several or all pharmaceutical options and were considered difficult to treat with a high headache burden.

### SPG microstimulator system

The SPG Microstimulator System is designed to fit in the facial anatomy with an integrated lead placed proximate to the SPG. The microstimulator communicates with a handheld remote control by radio-waves, is inductively powered and contains no battery. Using the remote control, patients apply on-demand SPG stimulation to treat individual cluster attacks and were advised to treat these for at least 15 min as soon as they were felt. Patients were also permitted to use SPG stimulation prophylactically without any sense of an impending or ongoing attack.

### Data collection

Response to SPG stimulation during each treated attack was collected prospectively in an electronic headache diary incorporated into the remote control. Pain scores were reported using the Categorical Pain Scale (CPS; 0 = none, 1 = mild, 2 = moderate, 3 = severe, 4 = very severe). Headache pain was reported just prior to stimulation use and headache pain and acute medication were reported immediately following stimulation.

Average attack frequency, laterality and acute and preventive medication use were collected retrospectively at each clinic visit, with patients asked to recall their attack frequency and acute medication usage over the prior 4 week period. Headache Impact Test (HIT-6) and Short Form Health Survey (SF-36v2) questionnaires were completed at baseline and quarterly during the first year. Following microstimulator insertion, a patient experience questionnaire was completed quarterly. Adverse events were collected throughout the study.

### Outcomes and analyses

Per protocol analyses included percent change in attack frequency, percent of attacks achieving effective therapy, the number of patients achieving acute and/or frequency effect, changes in acute and preventive medication, characterization of HIT-6 headache disability and SF-36v2 quality of life and patient evaluation of therapy using the patient experience questionnaire. Due to the different nature of eCH and cCH additional frequency analyses were performed separately for chronic and episodic patients. Surgical outcomes, including insertion of the microstimulator within the pterygopalatine fossa, explants and lead revision rates were collected.

Change in the frequency of attacks was analyzed as the change in attack frequency from baseline (4 weeks prior to enrollment) to the 12 month visit (4 weeks prior to the 12 month visit) where Frequency Responders experienced at least a 50% reduction.

Analysis of the acute response included all attacks where SPG stimulation was applied and for which the patient entered diary responses through the 12 month study visit. An Acute Responder is a patient able to achieve effective therapy in at least 50% of attacks.

Acute effective therapy was defined in the protocol as pain relief (decrease in categorical pain scale (CPS) from 2 (moderate) or higher to 1 (mild) or 0 (none)) without the use of acute medications or pain freedom (decrease in CPS from 1 (mild) or higher to 0 (none)) without the use of acute medications as evaluated immediately following the cessation of SPG stimulation.

Furthermore, therapeutic responders were defined as patients who were either acute responders, frequency responders, or both. An additional post hoc analysis was performed to evaluate therapeutic response at the 75% and 30% levels.

Change in acute medication use compared to baseline was calculated for all acute medication uses (average number of uses per week), as well as separately for triptans and oxygen. Preventive medication changes were characterized to identify patients who stopped, decreased dose, remained off, or added/increased doses of preventive medications.

Headache impact improvements were considered clinically meaningful when scores improved by at least 2.3 units relative to baseline [13]. Quality of life improvements were considered clinically meaningful if either the physical (PCS) or mental component score (MCS) improved by at least 4 units relative to baseline [14]. Responses to a patient experience questionnaire were characterized. In 8 patients, who missed their 12 month study visit, any missing data for frequency, medication, or questionnaire data were imputed from the preceding 9 month study visit. HIT-6 data was incomplete in 5 patients and 6 patients had incomplete SF-36v2 data. These were omitted from the respective analyses. With regards to study visits and duration, 28 days were considered a month. Some patients completed their 3, 6 or 12 month visit earlier or later than scheduled (see results) based on the protocol defined visit schedule and visit windows.

### Statistics

For all quantitative comparisons against baseline (frequency, acute medication, headache disability, quality of life) a paired t-test was used to account for changes over time with *p* < 0.05 regarded as statistically significant. Results for stimulation usage for acute cluster headache pain, were analyzed using a generalized estimating equation with least squares means. Changes in use of medication were calculated as absolute changes using Wilcoxon signed ranks.

## Results

### Patient disposition, clinical characteristics, and surgical outcomes

**Fig. 1:**
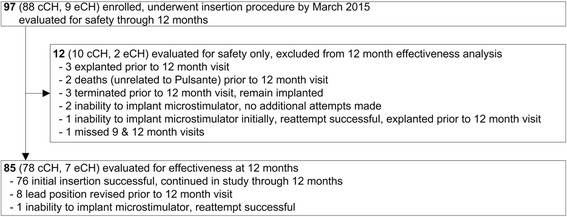
Patient disposition for the Pathway R-1 study 12 month interim analysis

Ninety-seven CH patients (88 chronic, 9 episodic) were enrolled at 12 centers (Germany: 10, Denmark: 1, Austria: 1) and underwent microstimulator insertion from September 2012 through March 2015 (Fig. 1, Table 1). None of these patients were included in previous studies of SPG stimulation. Four patients were not able to be implanted during the initial attempt, however two of the four underwent an additional insertion attempt and a microstimulator was successfully placed within the pterygopalatine fossa. Twelve patients did not complete the study through to the 12 month visit (Fig. 1). Two of these have some efficacy data available indicating non-response to therapy. The remaining 85 patients (78 chronic, 7 episodic) continued through the study to 12 months and are included in the present 12 month evaluation of effectiveness (average time from microstimulator insertion to 12 month study visit: 368 ± 42 days (range 245–475)). Of the 85 patients included in the final analysis at 12 months, 8 underwent lead repositioning prior to the 12 month time point. Seven of these were repositioned on the same day as the original surgery as post-op CT showed misplacement. The eighth patient was explanted 3 months after the original surgery and re-implanted 9 months later as lead placement was deemed to be suboptimal. Three patients (1 episodic, 2 chronic) had a frequency of 0 attacks/week at baseline. These patients were still implanted as they were very well characterized clinically, had CH for many years and logistics did not allow postponement.

**Table 1:** Baseline characteristics for the 97 patients included in the safety analysis. Data are presented as mean ± SD (range). In case of missing data, the number of evaluated patients is given in parentheses

Baseline characteristics (*n* = 97 evaluated for safety)	
Age	46.6 ± 11.7 (22–75)
Gender	71 Male/26 Female
Cluster headache subtype	88 Chronic/9 Episodic
Laterality of headache	56 Left/41 Right
Cluster headache duration (years)	13.4 ± 9.0 (1–44) (*n* = 95)
Cluster attack frequency (per week)	25.6 ± 20.9 (0–96) (*n* = 95)
HIT-6 score at baseline	64.4 ± 6.4 (41–78) (*n* = 92)
Preventive medications at baseline	28% (27/97) used no preventive medications 72% (70/97) used preventive medications Of these: 48% (47/97) used verapamil as monotherapy or part of polytherapy, 24% (23/97) used other medications^a^

*HIT-6* Headache Impact Test

^a^Topiramate, valproic acid, gabapentin, lithium carbonate, melatonin, methysergide, ergotamine and indomethacin

### Attack frequency, chronic patients

Fifty-five per-cent (43/78) of the cCH patients were frequency responders, defined as experiencing at least a 50% reduction in attack frequency. For these, frequency was significantly reduced at 12 months compared to baseline (Table 2).

**Table 2:** Change in attack frequency (mean ± SD (range)) between baseline and 12 months post-microstimulator insertion

Analysis	All patients		Chronic patients only		Episodic patients only	
	Baseline frequency (attacks/week)	12 month frequency (attacks/week)	Baseline frequency (attacks/week)	12 month frequency (attacks/week)	Baseline frequency (attacks/week)	12 month frequency (attacks/week)
All patients	25.2 ± 20.3 (0–96)	14.4 ± 17.9 (0–70)	24.3 ± 18.2 (0–84)	14.9 ± 18.1 (0–70)	34.3 ± 37.7 (7–96)	9.1 ± 13.6 (0–30)
	*p* < 0.0001, *n* = 85		*p* < 0.0001, *n* = 78		*p* = 0.1094, *n* = 7	
Frequency responders	26.7 ± 22.4 (3–96)	3.3 ± 6.3 (0–28)	24.7 ± 19.1 (3–84)	2.9 ± 5.4 (0–25)	30.0 ± 37.8 (7–96)	10.0 ± 14.8 (0–30)
	*p* < 0.0001, *n* = 48		*p* < 0.0001, *n* = 43		*p* = 0.0625, *n* = 5	

In the 78 chronic patients evaluated, 23 (29%) reported no attacks at the 12 month visit. For these cCH patients experiencing no attacks, the preventive effect manifested 150 ± 102 (range 8–390) days after microstimulator insertion. In all chronic patients, the average frequency at 3 months, 6 months, 9 months and 12 months was 14.9 ± 17.6, 14.9 ± 18.9, 14.2 ± 17.4 and 14.9 ± 18.1, respectively. At the 12 M visit 9 cCH patients experienced an increase in frequency of ≥ 50%.

Transitional therapies (oral steroids and greater occipital nerve (GON) blocks) were permitted at any time. Three of the 48 frequency responders (6%) received transitional therapies between microstimulator insertion and the 12 month visit. One patient received oral steroids at the 12 month visit, another had a GON block at 7 months post baseline, and a third a GON block 1½ months after microstimulator insertion. Of the frequency non-responders, six (16%) received GON blocks (0–6 months before 12 month visit), and two received oral steroids (6 and 9 months before 12 month visit).

### Acute effectiveness, episodic and chronic patients

Among all 85 patients, 13,600 attacks were treated with acute SPG stimulation (average 160 ± 245, median 82, range 0–1424 attacks per patient). Per protocol, 39% (5304/13600) of these attacks were effectively treated with SPG stimulation, with 26% (3534/13600) achieving pain freedom (Additional file 1: Figure S1). 32% (27/85) of patients were acute responders achieving effective therapy in at least 50% of their attacks. These acute responders were able to effectively treat 86% (4157/4811) of their attacks, with 57% (2750/4811) achieving pain freedom. Average SPG stimulation application for acute pain was 16.9 ± 12.1 min (0.02–127.3 min). Stimulation duration was not statistically different between attacks achieving effective therapy and those not (18.9 ± 15.7 and 15.7 ± 8.8 min, respectively, *p* = 0.5024).

### Therapeutic endpoints, chronic patients

As described above, patients using SPG stimulation experience both acute and frequency effects, and therapeutic response was therefore defined as experiencing either acute and/or frequency response. In the chronic patients, 65% (51/78) were considered responders per protocol (achieving effective therapy in at least 50% of attacks or experiencing a 50% reduction in attack frequency or both). Seventeen patients (20%) experienced both an acute and frequency response per the protocol. Nearly half of the chronic patients (47% (37/78)) experienced a very strong ≥ 75% response to SPG stimulation. 74% (58/78) of patients experienced at least a 30% therapeutic response.

### Acute and preventive medication use

**Fig. 2:**
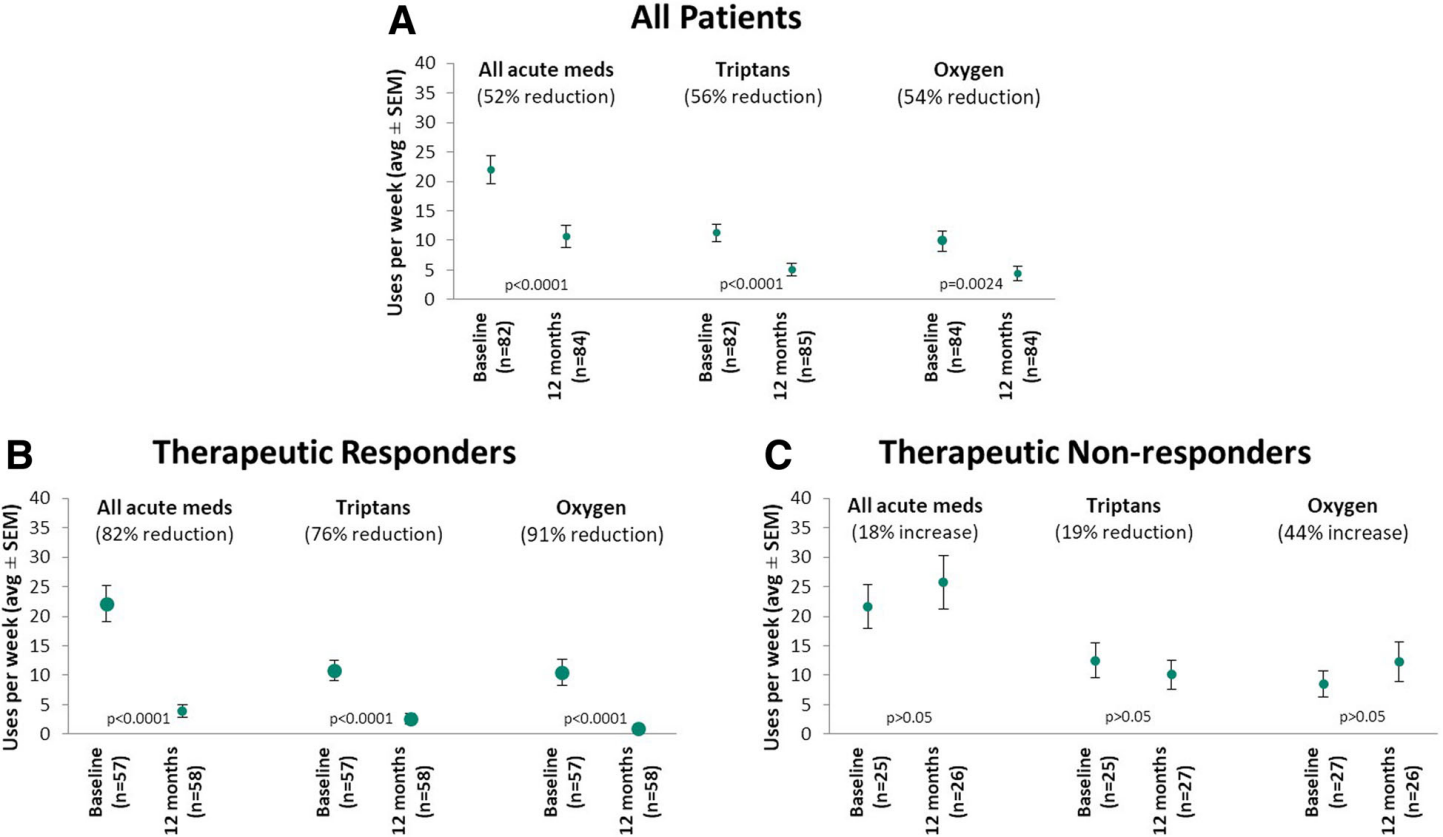
Acute medications at 12 months as compared to baseline for (**a**) all patients, (**b**) therapeutic responders and (**c**) therapeutic non-responders

Ninety-seven percent (82/85) of patients used acute treatments (triptans and/or oxygen) at baseline. All acute treatments were reduced by 52% from 22.0 ± 21.3 to 10.6 ± 17.6 uses/week (*p* < 0.0001, *n* = 82), triptan usage was reduced by 57% from 11.3 ± 13.3 to 5.0 ± 9.9 uses/week (*p* < 0.0001, n = 82), and oxygen usage was reduced by 54% from 9.8 ± 15.4 to 4.5 ± 11.3 uses/week (*p* = 0.0024, *n* = 84) (Fig. 2). Among those using acute medications at baseline, 67% (52/78) reduced all acute treatments, 87% (59/68) reduced triptan use, and 79% (34/43) reduced oxygen use by ≥50% compared to baseline.

At 12 months, for chronic patients only, improvements in preventive medication use were noted in 74% (58/78) with 18 stopping all, 19 stopping some and/or decreasing the dose of others, 20 remaining off all, and 1 experiencing a clinical improvement (per clinical judgment) in preventive medication use at 12 months versus baseline.

### Episodic cluster headache patient outcomes

Seven patients with eCH were followed through the study and per protocol, two were acute responders. This study is not ideally suited to evaluate the frequency response in eCH patients, however, per protocol, 5 of these were frequency responders. These eCH patients also saw improvements in quality of life and headache impairment similar to chronic patients: HIT-6 baseline: 64.5 ± 6.7, 12 months: 54.6 ± 13.2, SF-36v2 PCS baseline: 39.7 ± 7.9, 12 months: 44.9 ± 12.9 and SF-36v2 MCS baseline: 29.7 ± 8.4, 12 months: 40.4 ± 13.2.

### Headache disability, quality of life, and patient surveys

Fifty-nine percent (47/80) of all the patients that completed HIT-6 at baseline, and 74% (39/53) of therapeutic responders were HIT-6 responders, experiencing a reduction in headache disability of at least 2.3 points. 67% (53/79) of all the patients that completed the SF-36, and 73% (38/52) of therapeutic responders, were SF-36v2 responders, experiencing an improvement in PCS, MCS, or both in the SF-36v2 survey. In addition, HIT-6, SF-36v2 PCS, and SF-36v2 MCS scores were significantly improved at 12 months compared to baseline (Table 3).

**Table 3:** Changes in headache disability (HIT-6) and quality of life (SF-36v2 physical component score (PCS) and mental component score (MCS)) from baseline to 12 months post-microstimulator insertion are provided (mean ± SD (range))

Analysis	HIT-6 headache disability		SF-36v2 quality of life – PCS		SF-36v2 quality of life – MCS	
	Baseline	12 months	Baseline	12 months	Baseline	12 months
All patients	64.3 ± 6.6 (41–78)	56.0 ± 11.1 (36–76)	40.4 ± 7.4 (23.9–57.6)	43.5 ± 10.1 (21.7–63.3)	32.2 ± 13.7 (6.0–59.7)	39.2 ± 15.0 (6.1–65.1)
	*p* < 0.0001, *n* = 80		*p* = 0.0062, *n* = 79		*p* = 0.0003, *n* = 79	
Therapeutic responders only	64.7 ± 6.9 (41–78)	53.2 ± 11.1 (36–74)	39.9 ± 7.7 (23.9–57.6)	45.2 ± 10.4 (22.3–63.3)	32.2 ± 12.6 (14.4–57.7)	40.8 ± 14.8 (6.1–65.1)
	*p* < 0.0001, *n* = 53		*p* = 0.0001, *n* = 52		*p* = 0.0005, *n* = 52	

Differences were statistically significant for all patients, as well as for therapeutic responders

Seventy percent (56/80) of all patients who completed the questionnaire and 86% (48/56) of therapeutic responders rated the Pulsante™ system as good or very good. 81% considered the sensation of stimulation tolerable, 83% found the inserted device comfortable or did not feel it, 81% found surgery effects tolerable, 86% would recommend the therapy to someone else, 80% would make the same decision again, and 76% found SPG stimulation useful for treating their attacks.

### Safety

The occurrence of side-effects in this population was similar to previously published data [12] on this issue, which typically included sensory disturbances, post-operative pain and swelling (Additional file 2: Table S1). Briefly, 73% of patients experienced such postoperative sequelae and these were generally considered mild to moderate and resolved within 2–3 months (average: 68 days for resolved events). There were no cases of device/lead migration after correct placement. There were no cases of lead fracture. Eight patients underwent lead revision after initial insertion due to suboptimal electrode placement.

## Discussion

In this prospective study of 85 eCH and cCH patients treated with an implantable neurostimulator, we have demonstrated the effectiveness of SPG stimulation for attack frequency reductions and acute pain relief. At 12 months 55% of chronic patients were frequency responders and 32% of all patients were acute responders. At the per protocol defined level of ≥50%, 68% of all patients were therapeutic responders to SPG stimulation. 67% (52/78) of patients were able to reduce the use of acute medications by at least 50% and clinical improvements in the use of preventive medications were observed in 74% of patients. These clinical improvements were reflected in improved headache disability in 59% of all patients and 74% of responders.

Compared to deep brain stimulation and occipital nerve stimulation, SPG stimulation elicits both acute and preventive responses as supported by the reduction of both acute and preventive treatments in this population. Other studies in chronic headache have employed a responder level of 30% [15, 16]. If such a responder level were applied to this population, 76% of patients would be considered responders. However, some problems remain. For example, how the preventive response should be interpreted, given the possibility of spontaneous fluctuations [17]. The response to SPG stimulation appears stable over time as demonstrated in a previous 24 month study [9]. However, the difficulty in interpreting this response is underlined by results in the cCH group where the majority experienced reductions in frequency but a small subset *did* see an increase in the number of attacks compared to baseline. Further, in episodic CH, the frequency response cannot be solely attributed to treatment as the bout may be ending of its own accord. Longer baseline prospective attack diaries may be helpful in this regard, but given the dreadfulness of the disease, are impracticable and indeed unethical as it could delay effective treatment.

Overall, the data presented here is very similar to previously published results of SPG stimulation [8–10] with one exception: The proportion of acute uses of the stimulator achieving effective therapy was lower than previously reported (39% vs. 65%). This difference may be attributed to acute non-responders choosing the SPG stimulator over other acute treatments, despite it not being effective in them. This hypothesis may be supported by the fact that in the acute responders, the number of acute uses achieving effective therapy was similar in this study to that previously reported (86% vs. 78%, respectively). SPG stimulation is uniquely useful to CH patients as it is not associated with the daily limit in the number of uses as triptans which is especially important to patients with more than 2 daily attacks. Nor does it have the impracticality and stigmatization of oxygen, especially in the context of maintaining a professional life. Given that the use of acute medication is significantly reduced using SPG stimulation, this method also has an economical advantage [18].

As stated, SPG stimulation provides acute relief in some, a preventive effect in others, or both. This makes characterizing the efficacy challenging as reflected in previous studies [8, 9] where the preventive effect was unanticipated. However, this registry study was specifically designed to capture both signals. In the highly dynamic field of neurostimulation no official guidelines exist and there are only very few long-term neuromodulation studies of relatively small populations of CH patients. It should be noted that for want of a specific CH tool for measuring headache impact, the HIT-6, validated for migraine, was used. Although interventions with steroids were allowed per protocol, we believe these to have played a minor part in the overall results as only 3 of the 48 frequency responders were treated in this manner. A recall bias cannot be excluded, as the attack frequency was self-reported retrospectively at each study visit. Also, while a frequency effect is apparent, the effect of preventive simulation, i.e. stimulating without pain, is still unknown. At current, no positive or negative predictors for efficacy of SPG stimulation have been identified therefore patients with no immediate acute effect should keep stimulating as a preventive effect may manifest at a later point. An obvious possibility may be an influence of highly variant facial anatomy; however, other mechanisms should not be excluded. Additionally, stimulation duration and timing may play a role and future studies should investigate this and possible predictive factors.

## Conclusion

In conclusion, SPG stimulation is an effective treatment for CH patients reducing their need for medications and improving quality of life and reducing headache impairment.

## Additional files

Additional file 1: Figure S1. Effectiveness (pain relief or pain freedom) in the treatment of acute attacks in all patients and acute responders. (JPG 15 kb)

Additional file 2: Table S1. Number of reported adverse events in the whole population (*n* = 97) from 0 to 365 days, total 336 events. (DOCX 13 kb)

## Abbreviations

cCH: Chronic cluster headache
CH: Cluster headache
CPS: Categorical pain scale
eCH: Episodic cluster headache
GON: Greater occipital nerve
HIT-6: Headache Impact Test
MCS: Mental component score
PCS: Physical component score
SF-36v2: Short Form Health Survey
SPG: Sphenopalatine ganglion stimulation
