# Supplementary figures and images for: Sphenopalatine ganglion stimulation for cluster headache, results from a large, open-label European registry
Source: J Headache Pain. 2018 Jan 18;19(1):6. doi: 10.1186/s10194-017-0828-9 (PMC5773459; doi:10.1186/s10194-017-0828-9)

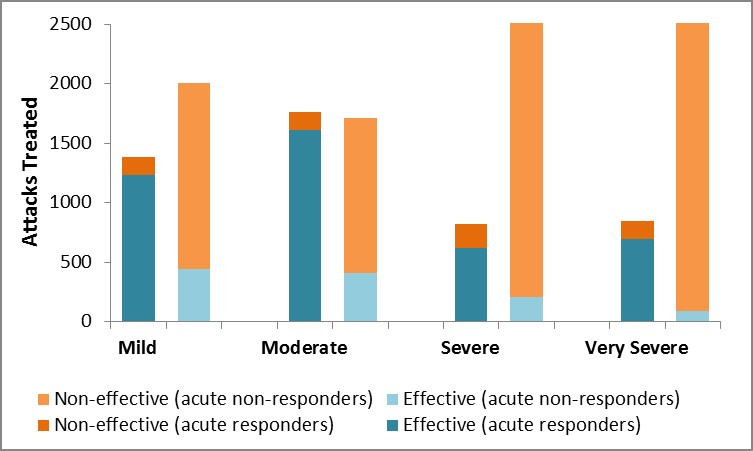

Supplement: Supplementary file 1 — Effectiveness (pain relief or pain freedom) in the treatment of acute attacks in all patients and acute responders. (JPG 15 kb) [file 10194_2017_828_MOESM1_ESM.jpg]
