# Supplementary material for: Sphenopalatine ganglion stimulation for cluster headache, results from a large, open-label European registry
Source: J Headache Pain. 2018 Jan 18;19(1):6. doi: 10.1186/s10194-017-0828-9 (PMC5773459; doi:10.1186/s10194-017-0828-9)
Supplement: Supplementary file 2 — Number of reported adverse events in the whole population (n = 97) from 0 to 365 days, total 336 events. (DOCX 13 kb) [file 10194_2017_828_MOESM2_ESM.docx]

Additional file 2: Table S1: Number of reported adverse events in the whole population (n=97) from 0-365 days, total 336 events.

| **Event Type** | **Patients (n=97)** | |
| --- | --- | --- |
|  | **# Events** | **% (97)** |
| Allodynia | 11 | 11.3% |
| Bleeding | 3 | 3.1% |
| Bone Damage | 2 | 2.1% |
| Device Failure | 2 | 2.1% |
| Dysesthesia | 5 | 5.2% |
| Epistaxis | 1 | 1.0% |
| Explant | 9 | 9.3% |
| Facial Asymmetry | 6 | 6.2% |
| Headache | 10 | 10.3% |
| Hematoma | 12 | 12.4% |
| Hyperesthesia | 7 | 3.1% |
| Hypoesthesia | 23 | 21.6% |
| Infection | 12 | 10.3% |
| Itching | 2 | 2.1% |
| Limited Jaw Movement | 11 | 10.3% |
| Numbness | 57 | 48.5% |
| Ocular Damage | 1 | 1.0% |
| Other | 57 | 34.0% |
| Pain | 27 | 23.7% |
| Paresis | 1 | 1.0% |
| Paresthesias | 18 | 15.5% |
| Swelling | 44 | 42.3% |
| Taste Alterations | 6 | 6.2% |
| Toothache | 2 | 2.1% |
| Trismus | 5 | 5.2% |
| Xerophthalmia (dry eye) | 2 | 2.1% |
